# Behavioral and psychological symptoms of dementia and their management

**Published:** 2009-01

**Authors:** Nilamadhab Kar

**Affiliations:** Department of Psychiatry, Wolverhampton City Primary Care Trust, Corner House Resource Centre, 300 Dunstall Road, Wolverhampton, WV6 0NZ, UK

**Keywords:** Behavioral and psychological symptoms of dementia, management of dementia, pharmacological management of dementia

## Abstract

**Purpose:**

Behavioral and psychological symptoms of dementia (BPSD) are an integral part of dementia syndrome. They increase morbidity and burden, affect quality of life and impact cost of care. This review aims to study the features of BPSD, their assessment and management.

**Collection and Analysis of Data:**

Literature of BPSD was searched in PUBMED and the relevant cross references were accessed.

**Conclusions:**

Available literature suggests that BPSD can manifest in multiple ways; the common components are of behavioral, affective, psychotic and somatic in nature. There are specific rating scales for assessing BPSD; however, there is need for developing cross-culturally validated instruments. Nonpharmacological interventions are preferred as first line, which mainly include environmental modification, social interactions, minimizing effect of sensory deficits and behavioral interventions. The judicious use of medications such as cognitive enhancers, atypical antipsychotics and antidepressants has been suggested in acute, emergent situations or when BPSD do not respond to other interventions. There is a research need to address etiologies, social and economic impact of BPSD, efficacy of pharmacological and nonpharmacological treatments and cost-effectiveness of these interventions.

## INTRODUCTION

Behavioral changes, paranoid delusions, hallucinations and long periods of screaming were described by Alzheimer in 1907 in his original case description of the disease.[1]

Behavioral and psychological symptoms of dementia (BPSD) are an integral part of dementia syndrome. Decline in emotional control or motivation, or a change in social behavior manifesting as emotional lability, irritability, apathy and coarsening of social behavior have been a part of diagnostic criteria for dementia.[2] Studies suggest that cognition and behavior are independent dimensions.[3] However, they influence each other; BPSD is associated with a more rapid rate of cognitive decline and greater impairment in activities of daily living;[4] and there are variations in severity of BPSD at different cognitive levels.[5] BPSD are often the reasons for the first contact with health professionals and hospitalization.[6] BPSD impact patient functioning and lead to premature transition to structured living environments and institutionalization.[7–10] They are a cause of concern and burden to the caregivers; and are often more difficult to cope with than cognitive changes.[11] These symptoms are a major cause of diminished quality of life for both patients and care givers.[12,13] BPSD contribute significantly to the overall costs of dementia care.[9,14,15] These noncognitive abnormalities which increase the morbidity of patients and burden of caregivers are mostly treatable. Their assessment and management are essential components of the treatment of dementia.[10] Periodic assessment of these symptoms can measure the effectiveness of interventions in dementia.[1,16] This review aims to study the features of BPSD, their assessment and management. Literature of BPSD was searched in PUBMED and the relevant cross references were accessed.

## PREVALENCE

In cross-sectional studies, reported prevalence of BPSD ranges from 50%-100%.[9,17] Lifetime risk of neuropsychiatric disturbances is nearly 100%.[18] In a study assessing severity, BPSD were severe in 36.6% of the patients, moderate in 49.3%, and mild in 14.1%.[19] Different types of BPSD have been reported in varying frequencies considering the type and degree of dementia, number of BPSD studied, environmental parameters and instrument used. Depression is usually the most common, which affects more than half of the patients.[20,21] However, apathy has also been found as the most common abnormality in some studies followed by anxiety and dysphoria.[5] Reported prevalences of some BPSD have been varied: agitation 55.9%, delusion 41.2%–63.0%, hallucinations 21%–26%, anxiety 35%–76%, sleep problems 26%–61%, apathy 70%.[20,22,23] Based on caregivers’ report, apathy, aberrant motor activity, dysphoria and anxiety were the symptoms that were most frequently reported, ranging from 46% to 74%.[24] Depending upon cognitive levels, variation in frequencies have been reported. BPSD were reported in 92.5% of the patients with a MMSE between 11 and 20, and in 84% of the patients with a MMSE between 21 and 30.[5]

## FEATURES OF BPSD

BPSD have myriad manifestations. There has been attempt to conceptualize these symptoms in various ways. Clusters comprising a psychotic syndrome and an affective syndrome have been frequently suggested.[25–27] Other major manifestations are in the areas of motor behavior, social interactions, speech, personality changes and somatic symptoms.

Inappropriate behaviors have been divided into four main subtypes: physically aggressive behavior, such as hitting, kicking or biting; physically nonaggressive behavior, such as pacing or inappropriately handling objects; verbally non aggressive agitation, such as constant repetition of sentences or requests; and verbal aggression such as cursing or screaming.[28]

BPSD in AD may reflect two largely independent pathophysiological processes: one associated with behavioral symptoms partly overlapping with attention and the other associated with psychological symptoms predominantly unrelated to cognitive function.[29]

There are marked regional and cultural variations in the type and frequency of the BPSD.[27,30,31] These differences may be explained by differing interpretation and administration of the measurement instruments, models of service delivery, availability of primary and secondary care services, health seeking behavior of patients and families, cultural influences, and knowledge, expectations and recognition of BPSD by professionals in primary and secondary care.

Motor behavior: Agitation manifests with restlessness, pacing, complaining, repetitive sentences, negativism, requests for attention, cursing and verbal aggression etc. Reported frequencies are between 24% and 48%.[32,33] Physical violence and hitting occurs in approximately 30% in Alzheimer’s dementia (AD).[33] Premorbid history of aggression, troubled premorbid relationship between caregiver and patient and multiple problems are predictors of aggressive behavior.

Almost a quarter of patients with AD report wandering. It has been found that elderly wanderers have language impairment, disorientation and hyperactivity compared to nonwanderers; on the other hand, wanders exhibit better social skills and are less withdrawn.[1] Symptoms like hyperorality (tendency to touch and examine objects with mouth), hypermetamorphosis (tendency to contact and touch every object in sight) and hyperphagia have been reported though uncommonly.[12,34]

Mood Disturbances: Depression is common, however it may not have a typical presentation - often there is a lack of sad or depressed affect or mood. The aphasic patient may not be able to articulate the subjective experience of being depressed.[35]

Depressive cognitions, death wishes are common. Other predominant mood disturbances are anxiety, fear, irritability, anger, etc. Emotional lability, explosive emotional outbursts, weeping or laughing are also seen.[36] Fatuous euphoria, elevated mood, inappropriate laughter have also been described.

Apathy, a syndrome of decreased initiation and motivation, decreased social engagement, emotional indifference, diminished reactivity and lack of persistence, affects over 70% of individuals with AD in the mild to moderate stages and over 90% of patients in the later stages; and is one of the most common neuropsychiatric symptom reported.[37] Distinction of the syndrome of apathy from depression can be made by the presence of dysphoria, hopelessness, guilt, self-criticism, suicidal ideation, sleep problems and appetite disturbances in the latter.[37] However, overlapping symptoms such as lack of interest in events and activities, anergia, psychomotor retardation and fatigue may make the differentiation difficult.

Personality change: Changes in personality are noticed with increasing passivity, coarsening of affect, decreased spontaneity, inactivity, feelings of insecurity, less cheerfulness and responsiveness. There are blunted individual characteristics or exaggerated premorbid traits. Loss of socialization, companionship, self-centered behavior, and irritability are also seen. Social behavior is marked by reduced initiative and drive, grossly insensitive behavior, lack of restraint, disinhibition, sexual misadventure, indolence, foolish jokes and pranks.[36]

Psychotic features: Psychotic features in patients with dementia are usually paranoid in nature. Some common ideas have been that some one is stealing things, being present in the room, living inappropriately in the home (phantom boarder), mishandling personal finances, planning to harm physically, etc. Other psychotic features are delusions of infidelity, hypochondriasis, zoopathy, dead relatives being still alive, erotomania, Capgras syndrome, believing television images are real, personal images in a mirror is a different person, misidentifying own home.[12,31,38–40] There are accompanying suspiciousness and misinterpretation.[12,31]

Other symptoms: Screaming is seen in almost 25% in nursing home residents.[1] It is often associated with toileting and bathing, severe cognitive impairment and high degree of dependency. Some patients have increased or decreased talk, muttering or mutism.[12] Sleep problems, e.g., nighttime and early morning awakenings are common. Sleep disturbance and daytime behavioral problems have been found to be related. High degree of dependency for activities of daily living, hygiene maintenance is marked.[1] Dependency for excretory functions and hygiene maintenance come as a burden to caregivers. Some patients remain bed ridden. Besides physical infirmity, various cognitive disabilities contribute to this dependency.[12]

## TYPES OF DEMENTIA AND BPSD

Some type of BPSD are more common in certain type dementia,[40] which suggest that a careful and systematic evaluation of BPSD is essential for characterizing disease-related features.[41] Type-specific BPSD, e.g., hallucination for Lewy body dementia (DLB), activity disturbances for frontotemporal dementia (FTD), anxiety and phobias for AD and affective disturbance for vascular dementia (VaD) have been identified. A strategy of targeting type-specific BPSD may be beneficial, such as environmental stimulus control for DLB patients who are prone to have hallucinations, design of a pacing path for patients with FTD who need support for symptoms of wandering, and emotional support for patients with VaD who are susceptible to depression.[42]

AD: In early stages of AD passivity, aspontaneity and reduced initiative have been described,[1] which may get interpreted as depression. Behavioral symptoms occur more frequently as the severity of dementia increase. Aggression, wandering, incontinence, and at least one symptom of Klüver-Bucy syndrome was found in 72%, namely misrecognition, apathy, rage, hypermetamorphosis, binge eating, sexual disinhibition or hyperorality in a study.[43]

DLB: Visual hallucinations are more commonly found in DLB than AD and are more complex, vivid and rapidly moving. Auditory hallucinations, persecutory delusions are also present. These fluctuate in their severity and become more in night. Depression is significantly more common in these patients.[36] In one study on DLB, anxiety was the most common BPSD (67.4%), followed by depression (61.9%), apathy (57.6%), agitation and sleep disorder (55.4%). Psychosis was present in half of the patients. These symptoms worsened over disease course and represented a core feature of the disease.[41]

VaD: As the capacity for judgment and a remarkable degree of insight is sometimes maintained for a long time in VaD, patient often reacts to the awareness of deficit by extreme anxiety and depression, which are seldom present in AD. Lability and explosive emotional outbursts, episodes of noisy weeping or laughing may occur on minor provocation, often without accompanying subjective distress or elation.[36]

Pick’s dementia: Changes of character and social behavior rather than impairment of memory and intellect have been the distinctive early features of Pick’s dementia. Fatuous euphoria or apathy, insensitive behavior, lack of restraint, and sexual misadventure have been seen. Delusions and hallucinations are relatively rare. Oral tendencies, overeating, tendencies to touch and seize objects within the field of vision appear early in contrast to AD where these appear late.[12]

Dementia due to Huntington’s disease: Emotional disturbance is a prominent premonitory feature.[12,36] BPSD are reported for some considerable time before chorea or intellectual impairment develops. Paranoid developments may be earliest manifestation, a change in personality: morose, quarrelsome, slowed, apathetic and neglectful persona is often observed. Sometime a florid schizophrenic or paraphrenic illness may be present for years before the Huntington’s disease becomes clearly apparent. Delusions of persecution, religiosity, reference and grandiosity are common. Depression, sometimes recurrent depressive psychosis, and anxiety may be marked from the outset.[12]

Creutzfeldt-Jakob disease (CJD): A prodromal stage is described in CJD which lasts for weeks or months characterized by neurasthenic symptoms. Fatigue, insomnia, anxiety, depression, mental slowness and unpredictability of behavior, auditory hallucinations and delusions are the usual complaints. Bouts of uncontrollable laughing and crying may be seen due to brain stem involvement.[12,36]

Alcoholic dementia: Profound social disorganization, deterioration of personality have been described in alcoholic dementia.[12]

## ETIOLOGY OF BPSD

Various theoretical models have been proposed to understand BPSD which basically guide the nonpharmacological intervention. They are: ‘unmet needs’ model, a behavioral/learning model, and an environmental vulnerability/reduced stress-threshold model.[28] Often the needs are not apparent to the observer or the caregiver, or the caregivers do not feel able to fulfill these needs. Unmet biological, social and psychological needs lead to discomfort and the distress, and in a background of impaired cognitive state and ineffective communication, these often manifest as BPSD.

Many problem behaviors are learned through reinforcement by caregivers or staff members, who provide attention when problem behavior is displayed. BPSD may arise possibly from the intensive approach to care where patients are stressed beyond their cognitive capabilities. It is assumed that dementia process results in greater vulnerability to the environment, and a lower threshold at which stimuli affects behavior. Persons with dementia progressively lose their coping abilities and therefore perceive their environment as more and more stressful,[44] which results in anxiety and inappropriate behavior.

Premorbid personality has also been linked to BPSD. Dementia accompanied by florid paranoid or affective symptoms is associated with abnormal personality earlier in life; persons with simple down ward course were found to have been more stable.[36] The relationship between premorbid neuroticism and BPSD is less straightforward, the former does not appear to be associated with depression in AD or predict an “affect” syndrome.[45] It has been suggested that some BPSD could be the consequence of both dementia and an undiagnosed comorbid bipolar spectrum disorder or a pre-existing bipolar diathesis pathoplastically altering the clinical expression of dementia.[7]

Underlying neurobiology of BPSD is still unclear. Agitation has been linked to dementia severity, brain-damaged state, specific psychiatric syndromes or symptoms, or specific types of psychopathology implicating frontal lobe dysfunction.[32,37] An involvement of some specific brain regions responsible for emotional activities (parahippocampal gyrus, dorsal raphe and locus coeruleus) and cortical hypometabolism have been suggested to contribute to BPSD.[9] AD-related apathy is thought to reflect the interaction between cholinergic deficiency and neuropathological changes in frontal brain regions.[37]

An imbalance of different neurotransmitters (acetylcholine, dopamine, noradrenaline, serotonin, GABA) has been proposed as the neurochemical correlate of BPSD.[9,46] Some evidence suggest that BPSD may result from increased norepinephrine (NE) activity and/or hypersensitive adrenoreceptors compensating for loss of NE neurons with progression AD.[47] Although homocysteine have been identified as an independent risk factor in AD, it does not seem to have an etiological role for BPSD.[48] In FTD, increased activity of dopaminergic neurotransmission and altered serotonergic modulation of dopaminergic neurotransmission is associated with agitated and aggressive behavior respectively.[49] Neurochemical mechanisms underlying the pathophysiology of BPSD are both BPSD-specific and disease-specific, which might have implications for more selective pharmacological treatments of BPSD.[49]

Dopamine transporter gene 3’-untranslated region variable number tandem repeat polymorphism (DAT1 3’-UTR VNTR) could play a role in susceptibility to irritability and aberrant motor behavior.[50] Similarly serotonin transporter SERT might have a minor role in the development of psychosis and aggressive/irritable tendencies.[51] However, these findings require replication in large well-characterized cohorts. Findings at present do not support a role for the APOE gene in BPSD.[52]

Sometimes agitation, irritation and many other BPSD symptoms may be due to an underlying medical cause. The patient may not be able to communicate the pain and distress in words. Pacing and restlessness may be due to drug side-effects. Delirium is a common trigger for BPSD.

## ASSESSMENT

Assessment of BPSD helps in mapping severity of morbidity, care needs and disease progression.[53] The assessment presents particular difficulty since both cognitive deficits and lack of insight may make the history from patients unreliable. Most scales in use for BPSD depend upon interviews with caregivers.

Scales that can specifically help in assessing BPSD include Apathy Evaluation Scale (AES),[54] Behavioural Rating Scale for Geriatric Patients,[55] Behaviour Pathology in Alzheimer’s Disease Rating Scale (BEHAVE-AD),[33] Behavioural Rating Scales for Dementia,[56] Cohen-Mansfield Agitation Inventory (CMAI),[57] Cornell Scale for Depression in Dementia (CSDD),[58] Frontal Systems Behaviour Inventory (FrSBe),[59] Neuropsychiatric Inventory (NPI),[60] Neuropsychiatric Inventory – Nursing Home version (NPI-NH),[61] the Apathy Inventory (AI)[62] and Behavioural and Psychological Symptoms Questionnaire (BPSQ).[17] There is a need to develop cross-culturally applicable scales and to evaluate translated versions of instruments for BPSD.[63] This will help in population-based epidemiological studies.

## MANAGEMENT OF BPSD

Management of BPSD involves psychological, behavioral, environmental, and pharmacological interventions. Treatment plan is individualized considering the need of the person, type of BPSD, previous response and pre-morbid experiences. Nonpharmacological intervention is the preferred initial method of intervention for BPSD, and combinations of interventions are usually suggested.[64] The key general elements in management of BPSD are clarification of target symptoms, ruling out delirium and comorbid major psychiatric diagnoses and creatively addressing possible social, environmental, or behavioral remedies.

### Nonpharmacological Intervention

#### Environmental modifications

The environment around the patient can be modified for a beneficial effect on the BPSD. Environments can be natural, enhanced, or reduced stimulation environments, depending upon the needs. A natural environment may mimic natural surroundings consisting of recorded songs of birds, babbling brooks or small animals, together with large bright pictures. An enhanced environment can be a simulated home environment with appropriate visual, auditory and olfactory stimuli which may decrease the chance of trespassing, exit seeking and other agitation behaviors.[28] Reduced stimulation environments are designed with camouflaged doors, neutral colors and pictures on walls, no televisions, radios, stereo players or ringing telephones, added with a consistent daily routine, and slow and soft speech for communication.[1,28] This modification helps decreasing agitation and use of restraint.

Calm, nontaxing environments, good lighting, prominent placement of frequently required objects, clocks, and calendars are helpful. Soft wall colors, nonskid flooring, contrast between the wall and floor, handrails are useful. A wanderer’s lounge where agitated patients can move safely may prevent wandering away. When the person is capable of going outside, access to outdoor area results in decreased agitation. Environment can be modified by installing adequate daytime lighting to improve sleep patterns in patients with disturbed sleep wake cycles.[28]

Clear and repetitive instructions, visual direction to different rooms through color lines and pictures decrease confusion. The environment should be maintained without frequent change; there should be minimum change of caregivers or staff.[65]

### Social interactions

One to one interaction for 30 min per day for 10 days has been found to be effective in decreasing verbally disruptive behavior.[66] Interaction in the mother tongue and regular intensive interaction help in reality orientation. Socialization can be increased by group activity, conjoint tasks and simple games.

In “simulated presence therapy,” audio or videos containing a relative’s portion of interaction is played, and pauses are given that allow the patient to respond to the relative’s questions. Displaying photos and names of family and friends in the patient’s living area is helpful.[28,65] Spending time with pets or having a pet at home (pet therapy) decreases agitation and verbal aggression.[67,68]

### Minimize the impact of sensory deficits

Corrective eyeglasses and hearing aids decrease risk of disorientation,[69] and one study reported a decrease in yelling.[28] Slow and repetitive explanations reduce confusion and agitation. Sensory stimulation or enhancement, by massage and touch, aromatherapy has been tried, but no significant impact has been reported. Data supporting the efficacy of aromatherapy are scarce; both positive and negative consequences have been reported.[70]

### Medical and nursing interventions

Prompt management of pain is helpful; periodic specific enquiries regarding pain are important in this regard. Practice of sleep hygiene, leading to adequate sleep in the night has been found to reduce agitation.[71] It has been suggested that agitation secondary to fatigue and circadian rhythm disturbances can be reduced by bright light therapy.[28] Although there is some evidence for influence of light therapy on sleep and circadian activity, it is not possible to conclude about efficacy of light therapy for BPSD or about the practicability in clinical settings and safety.[72] Music therapy has been shown to be effective to reduce BPSD in patients with moderate-severe dementia.[73]

### Behavioral interventions

Methods such as extinction, differential reinforcement and stimulus control have been used besides other behavioral interventions. Reinforcements include social reinforcements, food, touch, going outside, etc.[28] A positive impact of activities, consistent daily routines has been reported. Physical and social activities, watching and listening, along with day care have influenced BPSD, especially restless behaviors.[74] Adult day service can support with activities and more targeted behavioral strategies for patients and caregivers to manage BPSD.[75] Exercises, removal of restraints, and adequate rest help in reducing the inappropriate behavior. Spiritual and religious activities which have significance for the patients help to remain engaged in ritualistic observations.

### Staff and carers’ training

Training and education of the caregivers or staff about understanding BPSD and improving methods of addressing their needs have shown beneficial results. Examples of such programs are as follows: the CARE program (Calming Aggressive Reactions in the Elderly),[76] the NACSP (Nursing Assistant Communication Skill Program),[77] abilities-focused program of morning care, and training on activities of daily living (ADL).[65] One of the learning points is communicating clearly, which helps the patients to understand their environments, and this leads to less frustration, anger and aggression.

### Pharmacological Intervention

It is suggested that environmental and behavioral techniques for BPSD should be tried first;[37,78] and only in emergent situations or when the nonpharmacological methods have failed should medications be deployed.[79] There are many concerns for the medicinal management in elderly and ‘not every thing can be treated with medications’.[35] However, medicines are frequently used, and in fact often they compliment the environmental and behavioral interventions. Safe treatment of BPSD depends upon: differentiating medical from psychiatric causes of patient’s distress, using drugs as adjuncts to psychosocial treatments and to start low and go slow when titrating dosages.[80] Various classes of medications are used; cognitive enhancers, antipsychotics, mood stabilizers, antidepressants are common.

### Cognitive enhancers

Cholinesterase inhibitors (ChEIs), particularly rivastigmine, can delay the onset and reduce the severity of BPSD, and decrease the requirement for antipsychotic and other psychotropic medications.[81] Evidence suggests that they may be more appropriate for the control of chronic (over weeks to months) mild-to-moderate BPSD.[82] Findings support the view that ChEIs should be considered as first-line treatment of the cognitive and psycho-behavioral symptoms of both AD and DLB.[83] Rivastigmine has been found to improve BPSD in a number of studies[83–85] and suggested as a well-tolerated treatment option for improving or preventing psychotic and nonpsychotic symptoms associated with AD.[86] However, some studies have found no or limited effects on behavior.[87]

Efficacy of donepezil in reducing a range of BPSD, e.g., agitation, apathy, anxiety, delusions, depression, disinhibition and irritability has been reported in RCTs over 4–24 weeks.[88,89,90] Additional evidence suggests that donepezil use is associated with a reduced need for psychotropic medications (e.g., antidepressants) in patients with AD.[91] Acetylcholinesterase inhibitors should be considered for the treatment of BPSD before neuroleptic treatment is instituted in AD patients with low levels of irritability and agitation.[92]

Galantamine was found well-tolerated and effective for improving BPSD in one study;[93] however, another study found no change, while placebo patients worsened.[94] Studies involving memantine report both no change.[95] and stabilization of behavioral issues.[97] Memantine augmentation of donepezil therapy resulted in a reduction in behavioral disturbances in one report.[96]

### Antipsychotics

Antipsychotic medication for BPSD are recommended for short-term treatment of severe symptoms associated with considerable distress or serious risk.[78] Increased risk of mortality as a result of cardiovascular or infectious events associated with certain atypical antipsychotics is a concern.[97,98] However, some authors highlight the negligible rate of adverse effects and high rate of efficacy with respect not only to BPSD but also to cognitive and functional domains.[99] In expert opinions, atypical antipsychotics were preferred for treatment of most BPSD.[100] It is interesting that improvement in many BPSD e.g. agitation, aggression or other behaviors with antipsychotic medication treatment may not depend on distinct antipsychotic effects. In contrast, there is preliminary evidence that delusions and hallucinations may respond to treatment with medications outside the antipsychotic class.[101]

There are many placebo controlled RCTs and head-to-head studies supporting tolerability and the efficacy of risperidone in treatment of BPSD.[102–107] The overall efficacy of risperidone was judged as excellent by the general practitioners and caregivers in about half the patients.[19] Variety of BPSD have been treated with risperidone, which includes psychosis, affective symptoms, anxiety and phobias, aggression, agitation, irritability, sleep disturbances.[102,106,108]

It has been noted that low-dose haloperidol and risperidone were well tolerated and associated with reductions in the severity and frequency of BPSD; though risperidone may have a more favorable risk-benefit profile.[109] However, another study has found risperidone as significantly more effective than haloperidol in treating wandering, agitation, diurnal rhythm disturbances, anxiety regarding upcoming events, other anxieties, physical sexual advances, pacing and aimless wandering, intentional falling, hoarding things, performing repetitious mannerisms, repetitive sentence or questions, complaining and negativism.[110] Haloperidol does decrease the aggression,[111] but there is insufficient evidence demonstrating its effectiveness in controlling agitation.[112]

Superior efficacy of olanzapine over placebo has been reported in RCTs for treatment of BPSD.[113] Injectable olanzapine has also been used to control agitation;[79] it was found to be superior to placebo at 2 and 24 h.[114] Study findings suggest that olanzapine could be a safe and effective treatment even for elderly population in suitable doses and receiving the adequate follow-up.[115] However, there are reports which suggest that the overall differences in cognitive changes in patients treated with olanzapine or placebo were small and insignificant, negative effects on cognition in some patients could not be excluded.[116]

Quetiapine has been found effective in controlling BPSD with favorable adverse-event profiles.[117,105] There are reports of decrease in delusion and aggression in AD[118] and psychosis and agitation in DLB with quetiapine.[119] However, there are studies where quetiapine did not significantly improve psychosis scores.[120]

The information regarding ziprasidone, aripiprazole and clozapine are scanty. A retrospective study reported decreased agitation by ziprasidone, which was safe and effective in treating psychosis associated with dementia.[80] Aripiprazole compared with placebo had significant improvement in Brief Psychiatric Rating Scale psychosis subscale score.[80]

### Mood stabilizers

Many mood stabilizers have been used for BPSD with variable result. A recent attempt at conceptualizing at least some of the BPSD as underlying bipolar spectrum disorder[7] may give higher credence for use of mood stabilizers. Although clearly beneficial in some patients, anticonvulsant mood stabilizers cannot be recommended for routine use in the treatment of BPSD at the present time.[121]

Preliminary data suggest that valproate may be effective and safe for the treatment of agitation associated with dementia;[79,122] however, there were inconclusive results too.[123,124] Further research is needed to determine the efficacy of divalproex sodium for BPSD.[123]

There are only a few studies involving carbamazepine; and the results suggested both improvement.[125] and no change in BPSD.[126] Controlled studies involving lamotrigine, gabapentin, and topiramate are not available. In a case report, gabapentin has been found as a reasonable alternative therapy for BPSD which do not respond to conventional agents.[127]

### Antidepressants

Antidepressants are frequently used as depression is common in dementia. However, nonpharmacological intervention should be the first step, particularly for milder depression considering the safety advantage.[21] Studies suggest AD patients tolerate SSRIs better than tricyclic antidepressants (TCA). Low doses of sertraline or citalopram has been suggested as the most appropriate to begin with.[21]

Sertraline as an adjunct to donepezil has shown higher response rate compared to placebo.[128] A study comparing sertraline and haloperidol found significant improvement in both with no difference.[129] Paroxetine has led to improvement in verbal agitation in dementia patients.[130] Citalopram has showed significant benefit compared to placebo for agitation, lability and psychosis.[131] Case reports suggest fluvoxamine to be effective in the control of BPSD with AD.[132]

Case series, open trials and naturalistic follow-up studies suggest benefit of trazodone in BPSD.[133] Symptoms of irritability, anxiety, restlessness and depressed affect have been reported to improve in some cases. Trazodone is favored for sleep disturbance primarily and as second or third line for “mild” agitation.[79] In a comparative study with haloperidol, trazodone was better tolerated in addition to being equally effective for agitation.[101] Negative results have also been reported in a multi-center study contrasting trazodone, haloperidol, placebo and care giving.[134]

### Benzodiazepines

Benzodiazepines are often used as rescue medicines, though there is little evidence supporting their use or safety. Better efficacy of benzodiazepine compared to placebo has been reported.[114] However, considering high rates of side effects, benzodiazepines are advocated only for limited use, in acute circumstances and longer only when other agents have been proven ineffective.[79]

### Other approaches

Various other drugs have been tried, e.g., dopaminergic drugs, such as amantadine and bupropion, for apathy in AD; psychostimulants such as methylphenidate and dextroamphetamine for apathy; propranolol for aggression; and buspirone for mild agitation associated with anxiety or irritability.[65] However, clinical observation indicates that the duration of positive effect may be short lived and the results are not definitive.[65,79] Electroconvulsive therapy has been suggested for dementia patients with treatment-resistant severe depression especially when suicidality, dangerousness or violent thoughts are there.[21]

## CONCLUSION

BPSD are a part of dementia syndrome and cause of higher morbidity and poor quality of life for patients and carers. Assessment and management of BPSD is an essential part of dementia treatment. Behavioral and environmental interventions are recommended as first-line treatment, followed by judicious addition of medications. Cognitive enhancers, atypical antipsychotics and antidepressants are beneficial. There is research need to address cross-culturally applicable methods for assessment, underlying biological and psychological etiologies, social and economic impact of BPSD, efficacy of pharmacological and nonpharmacological treatment and cost-effectiveness of these interventions.
